# Supplementary material for: A ubiquitination-related risk model for predicting the prognosis and immunotherapy response of gastric adenocarcinoma patients
Source: PeerJ. 2024 Jan 31;12:e16868. doi: 10.7717/peerj.16868 (PMC10838090; doi:10.7717/peerj.16868)
Supplement: Supplemental Information 2 [file peerj-12-16868-s002.docx]

Supplementary methods

The primers of RNF144A and β-actin were designed with primer 5 software. β-actin (F ACCCTGAAGTACCCCATCGAG; R AGCACAGCCTGGATAGCAAC) has a product length of 224 bp. RNF144A (F TGTGATCTGGCATCGGACAC; R AAAGCGGAGTGGTTTCCCAA) has a product length of 156 bp. Cells were taken at time intervals of 0 h, 24 h, 48 h, and 72 h. The supernatant was then removed, and 100 L 10% CCK-8 solutions were added to each well. The cell supernatant containing CCK-8 was added to the 96-well plate after continuing to incubate it at 37°C with 5%C02 for an additional hour. The absorbance (OD) values at 450 nm were examined using the Bio-Tek enzyme label with four triplicate wells in each group. MKN-7 were separated into three groups after siRNA transfection: NC, si-RNF144A-1, and si-RNF144A-2. For EdU labeling, 50 M of EdU culture media was added. Apollo staining, DNA staining, and picture capturing were all done after cell immobilization. MKN-7 were separated into three groups after siRNA transfection: NC, si-RNF144A-1, and si-RNF144A-2. The upper compartment was filled with 100 l of Matrigel. To the lower compartment was put 500 L of DMEM containing 10% FBS. In the upper chamber, cultures of MKN-7 were grown. To fix the migrating cells, 4% paraformaldehyde was used. Crystal violet 0.1% was used to label the migrating cells. MKN-7 were separated into three groups after siRNA transfection: NC, si-RNF144A-1, and si-RNF144A-2. In the lower chamber, cultures of MKN-7 were grown. THP-1 cells were polarized into M0 cells after being treated with Phorbol 12-myristate 13-acetate (PMA) at 320 nM for 6 hours at 37 ° C. IL-4 and IL-13 were added, and the mixture was incubated at 37 ° C for 18 hours. A polarized M2 macrophage was observed under a microscope and captured on camera. The upper compartment was used to cultivate M2 macrophages. In order to fix the migrating cells, 4% paraformaldehyde was used. Crystal violet 0.1% was used to label the migrating cells.


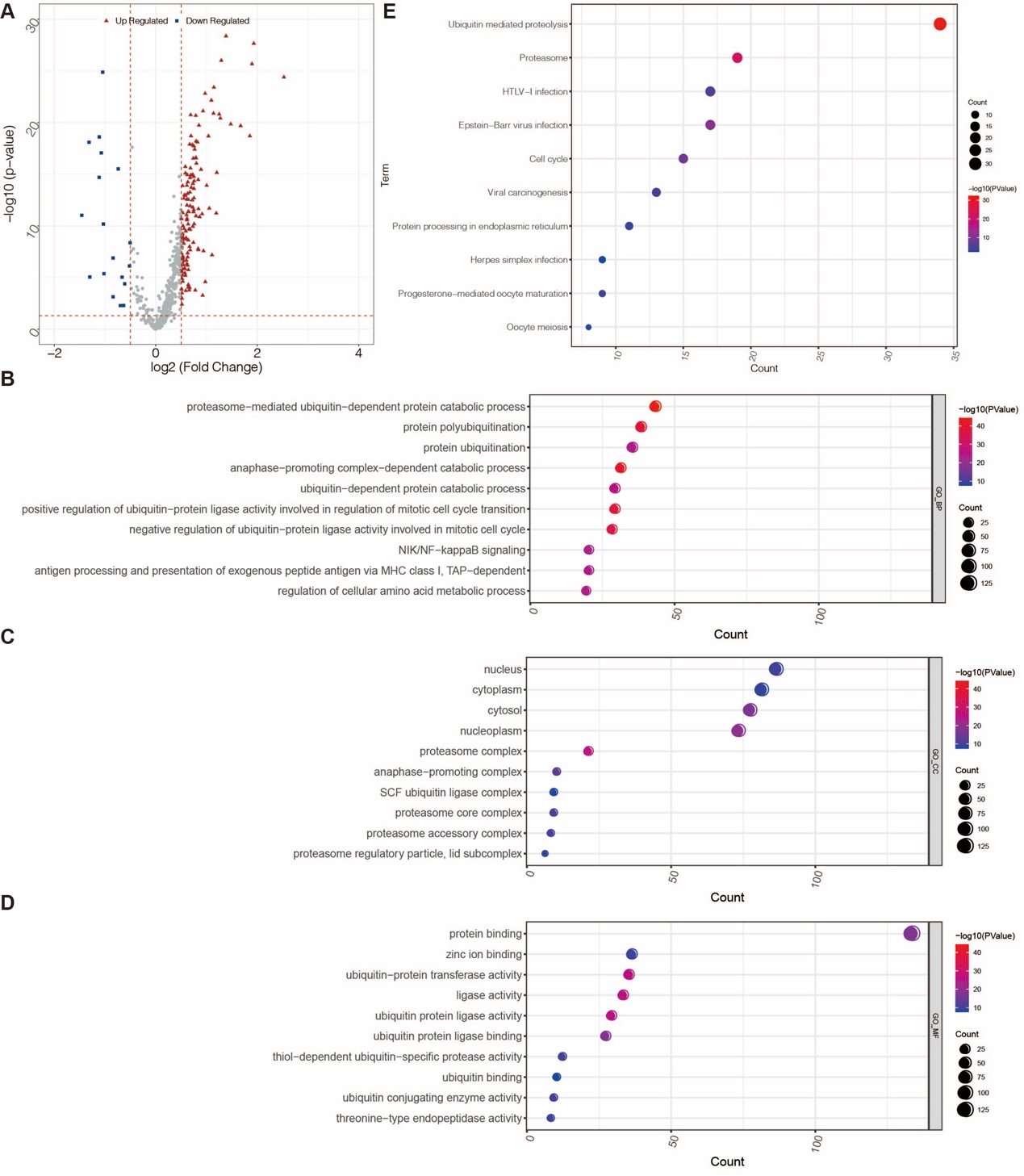


Figure S1. Analysis of the STAD-related DE-URGs A. Volcano plot showed the DE-URGs between the STAD group and the normal group. B. Enrichment analysis on the DE-URGs based on GO-BP terms. C. Enrichment analysis on the DE-URGs based on GO-CC terms. D. Enrichment analysis on the DE-URGs based on GO-MF terms. E. Enrichment analysis on the DE-URGs based on KEGG terms.


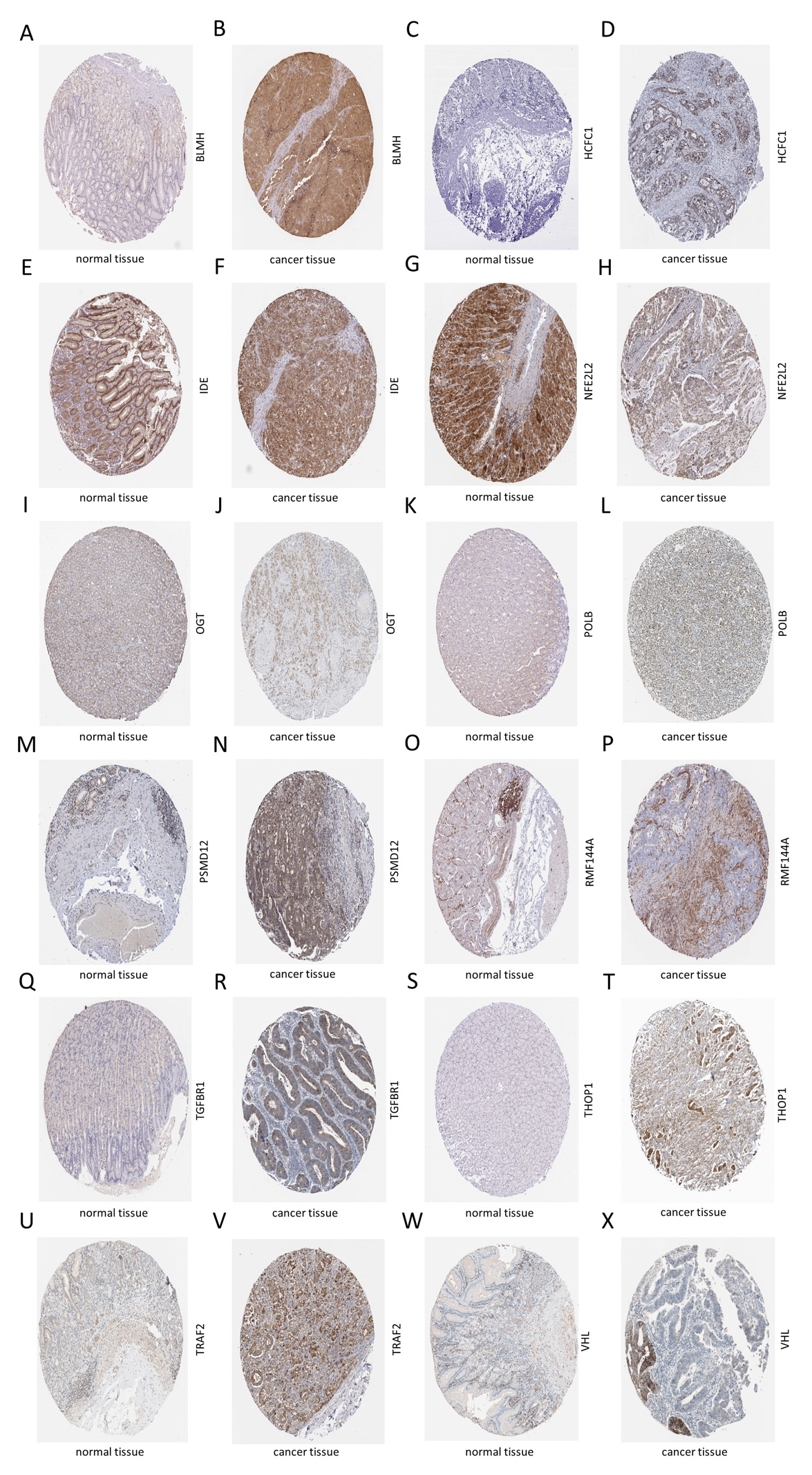


Figure S2. Representative images of IHC staining for the protein expression of 13 genes from the Human Protein Atlas database.


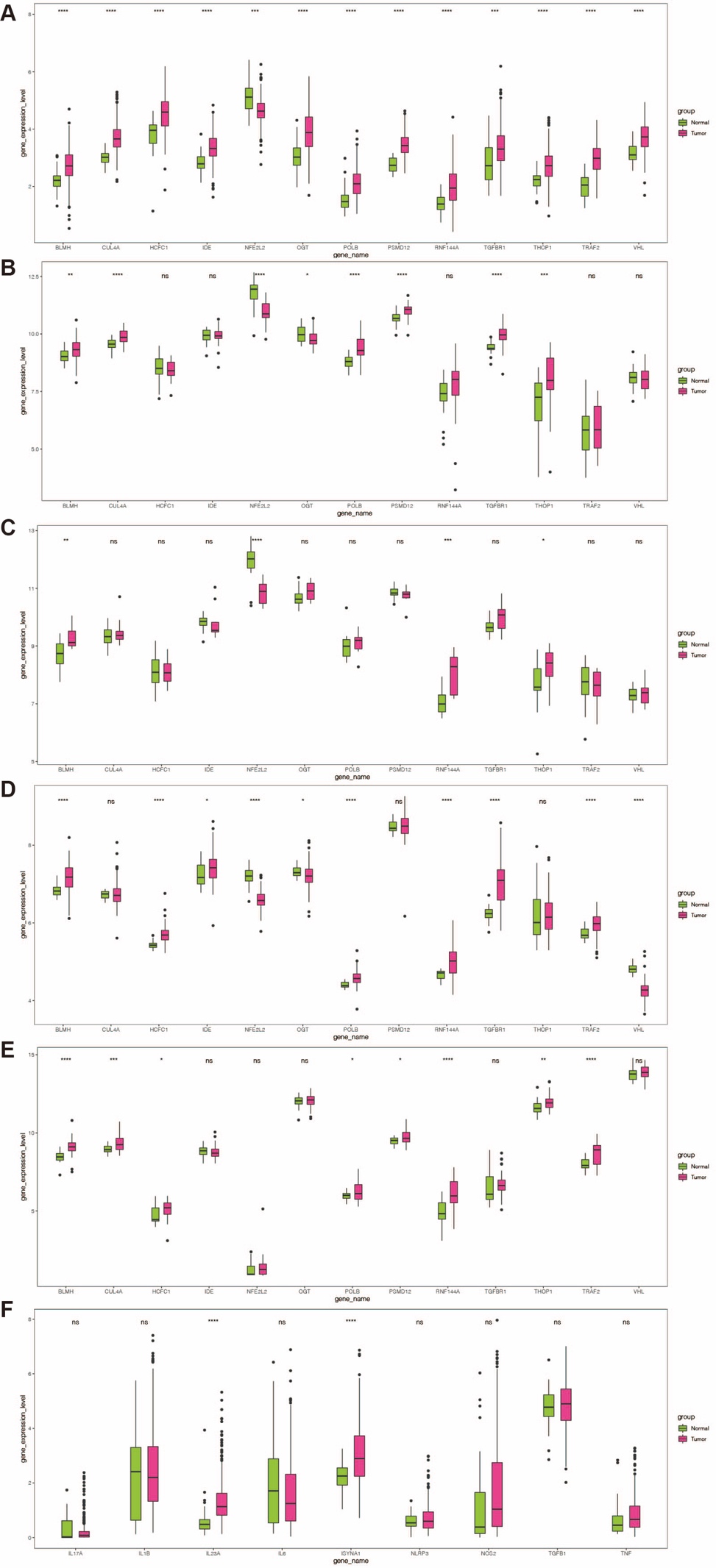


Figure S3. Expression verification for the 13 DE-URGs. A. Box plot demonstrated the varying presentations of model genes in the high-risk and low-risk groups in the TCGA dataset. B. Box plot demonstrated the varying presentations of model genes in the high-risk and low-risk groups in the GSE13911 dataset. C. Box plot demonstrated the varying presentations of model genes in the high-risk and low-risk groups in the GSE19826 dataset. D. Box plot demonstrated the varying presentations of model genes in the high-risk and low-risk groups in the GSE54129 dataset. E. Box plot demonstrated the varying presentations of model genes in the high-risk and low-risk groups in the GSE65801 dataset.


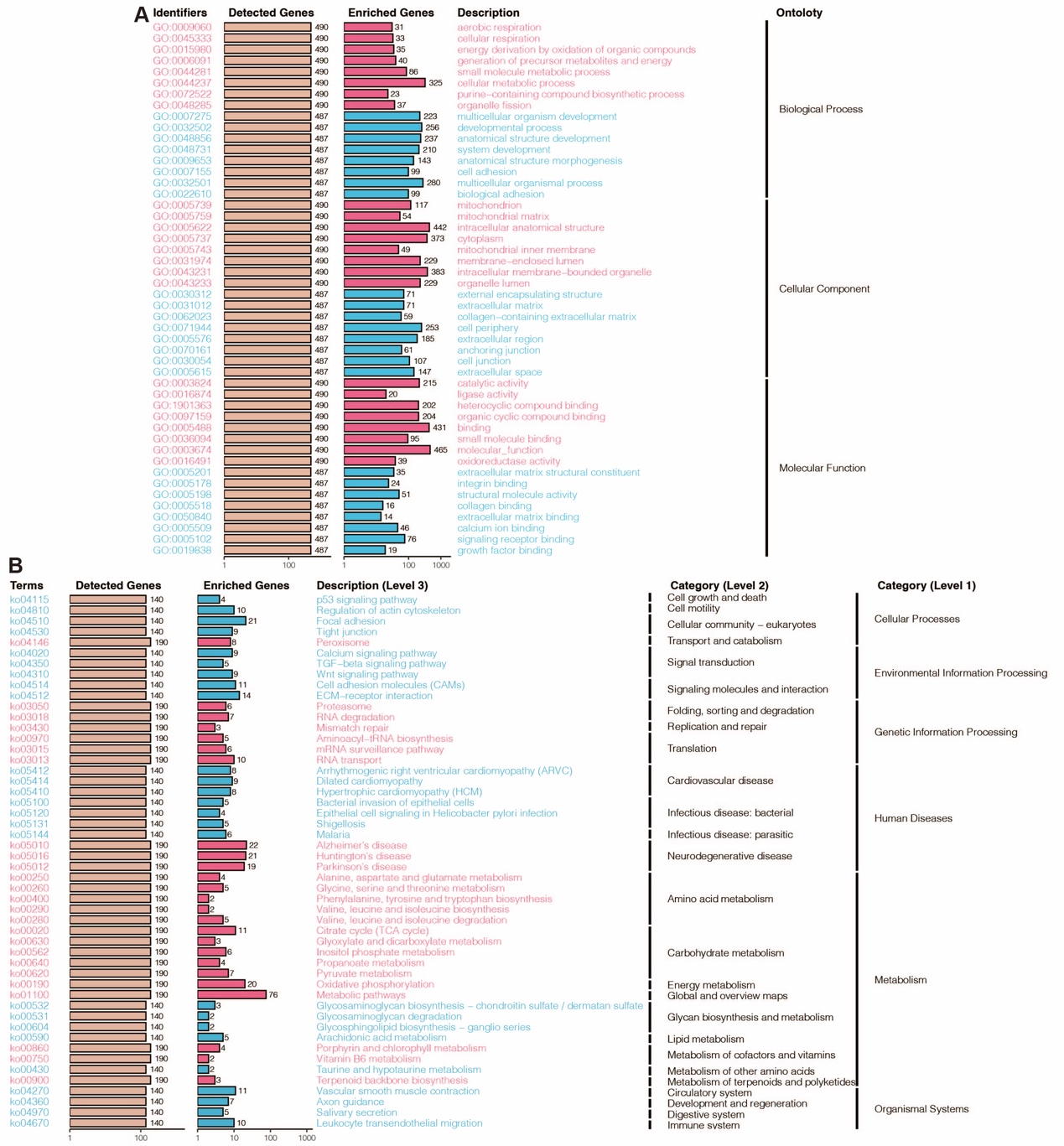


Figure S4. Function annotation of the enhanced prognostic signature. A. GO enrichment analysis. B. KEGG enrichment analysis.


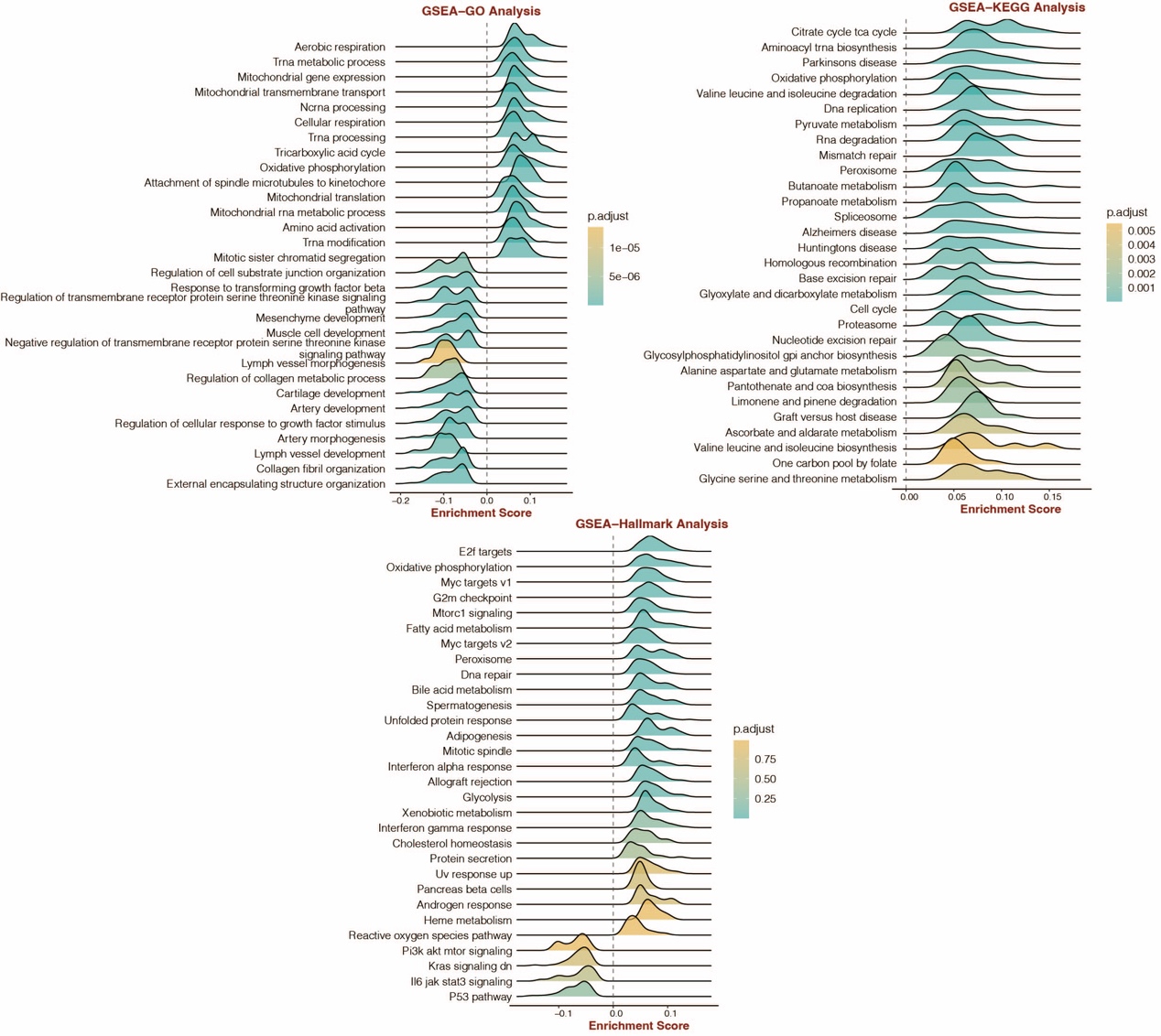


Figure S5. Function annotation of the enhanced prognostic signature. GO, KEGG, and HALLMALK-related GSEA analysis.
