# Supplementary figures and images for: A ubiquitination-related risk model for predicting the prognosis and immunotherapy response of gastric adenocarcinoma patients
Source: PeerJ. 2024 Jan 31;12:e16868. doi: 10.7717/peerj.16868 (PMC10838090; doi:10.7717/peerj.16868)

# qPCR

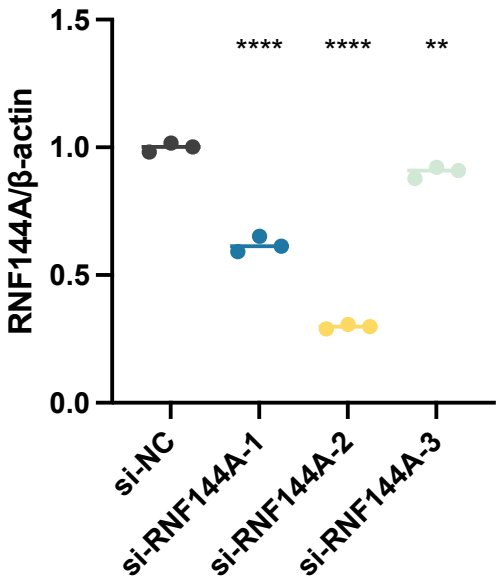

Supplement: Supplemental Information 4 — qPCR assay. [file peerj-12-16868-s004.zip › Figure 8A/qPCR.pdf]

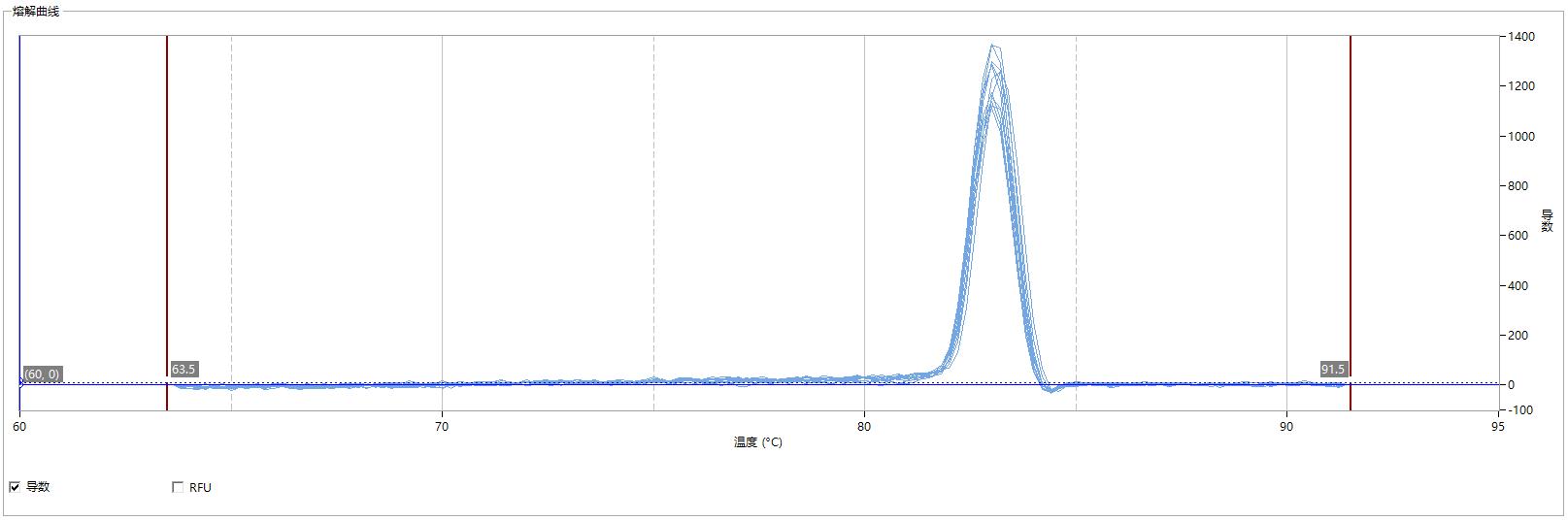

Supplement: Supplemental Information 4 — qPCR assay. [file peerj-12-16868-s004.zip › Figure 8A/actin-melting curve.jpg]

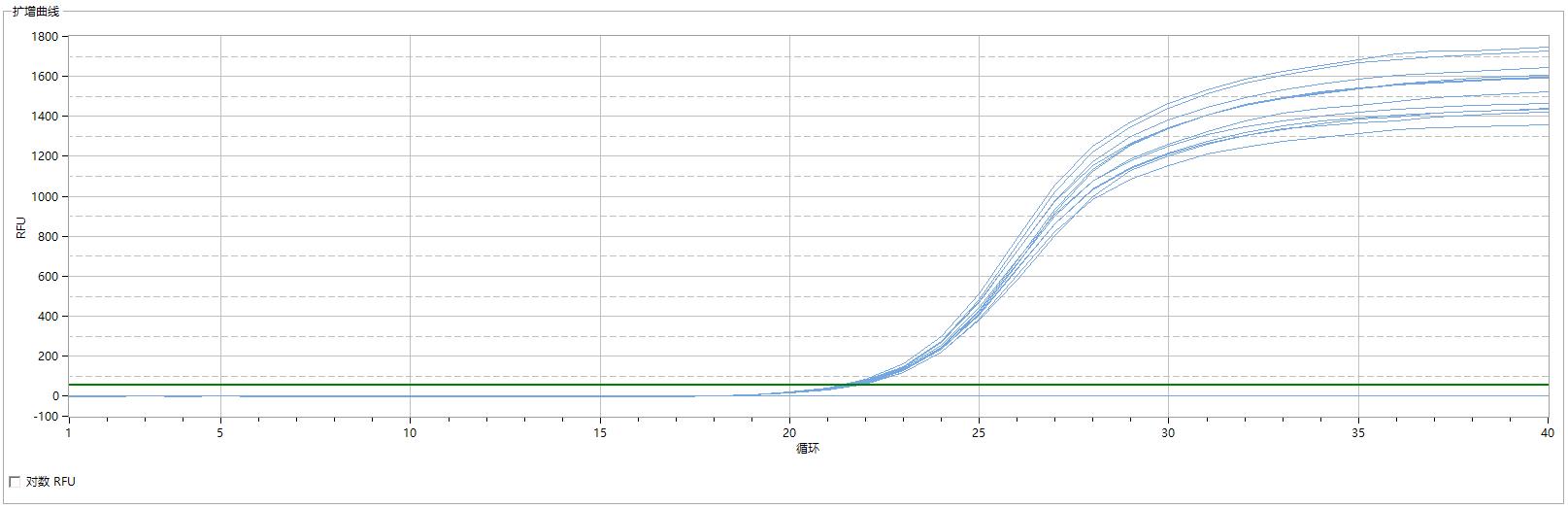

Supplement: Supplemental Information 4 — qPCR assay. [file peerj-12-16868-s004.zip › Figure 8A/actin-amplification curve.jpg]

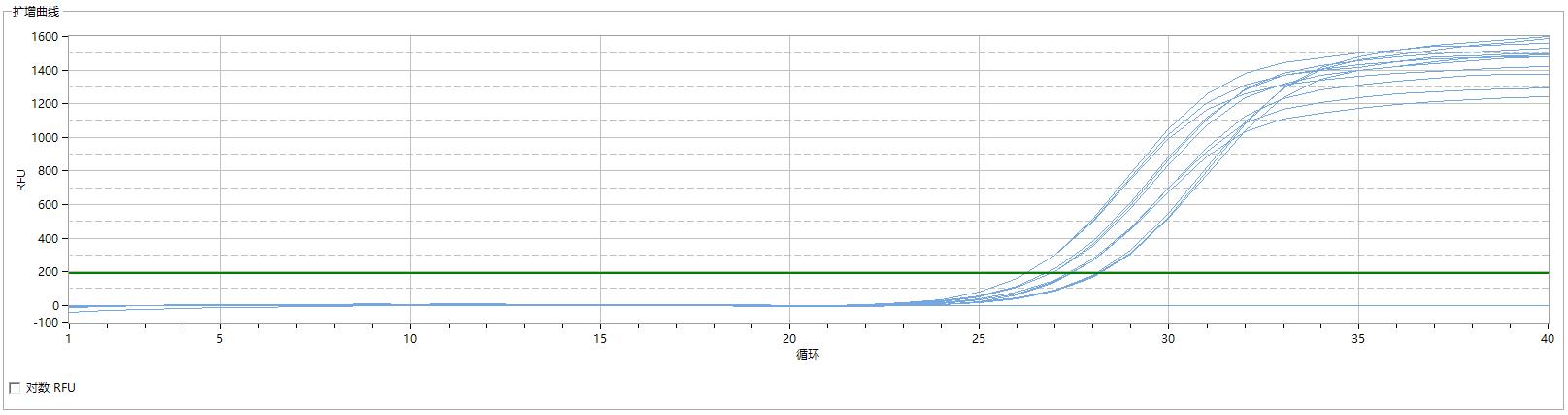

Supplement: Supplemental Information 4 — qPCR assay. [file peerj-12-16868-s004.zip › Figure 8A/RNF144A-amplification curve.jpg]

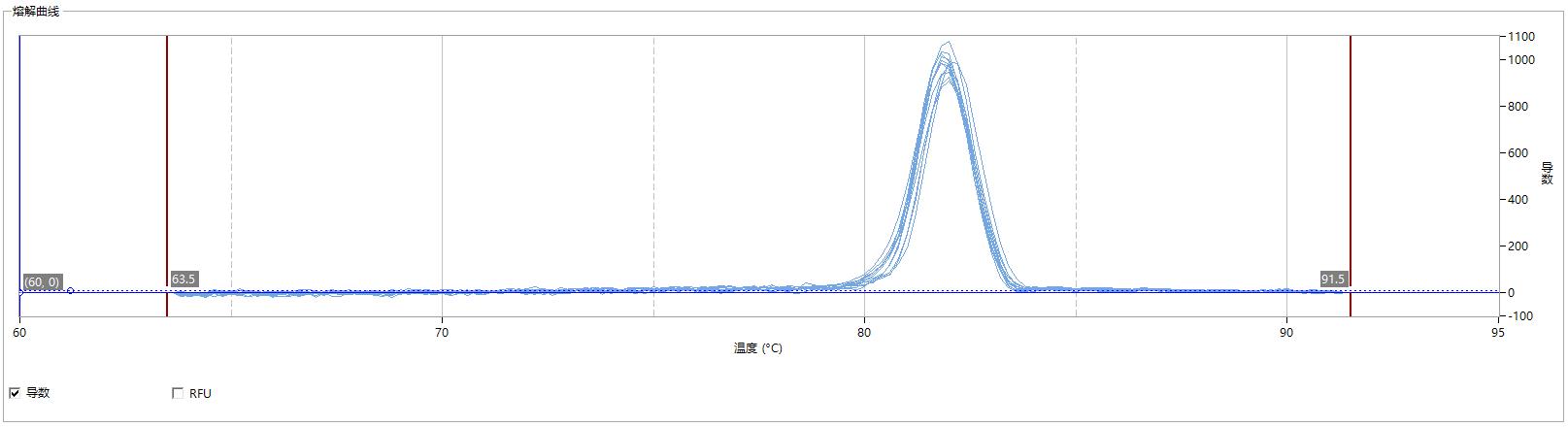

Supplement: Supplemental Information 4 — qPCR assay. [file peerj-12-16868-s004.zip › Figure 8A/RNF144A-melting curve.jpg]

# CCK8

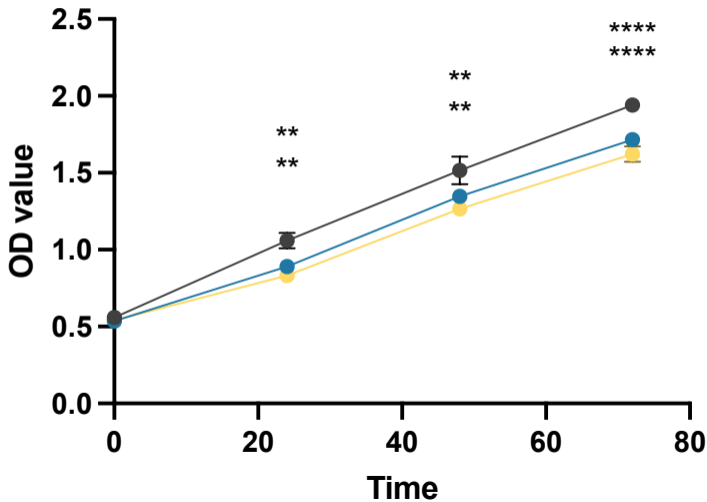

Supplement: Supplemental Information 5 — CCK-8 assay. [file peerj-12-16868-s005.zip › Figure 8B/CCK8.pdf]

# EdU

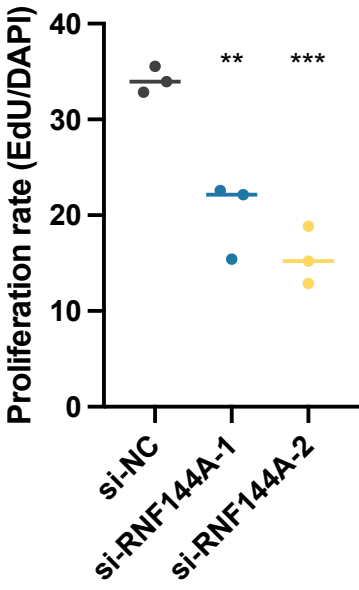

Supplement: Supplemental Information 6 — EdU assay. [file peerj-12-16868-s006.zip › Figures 8C and 8D/EdU.pdf]

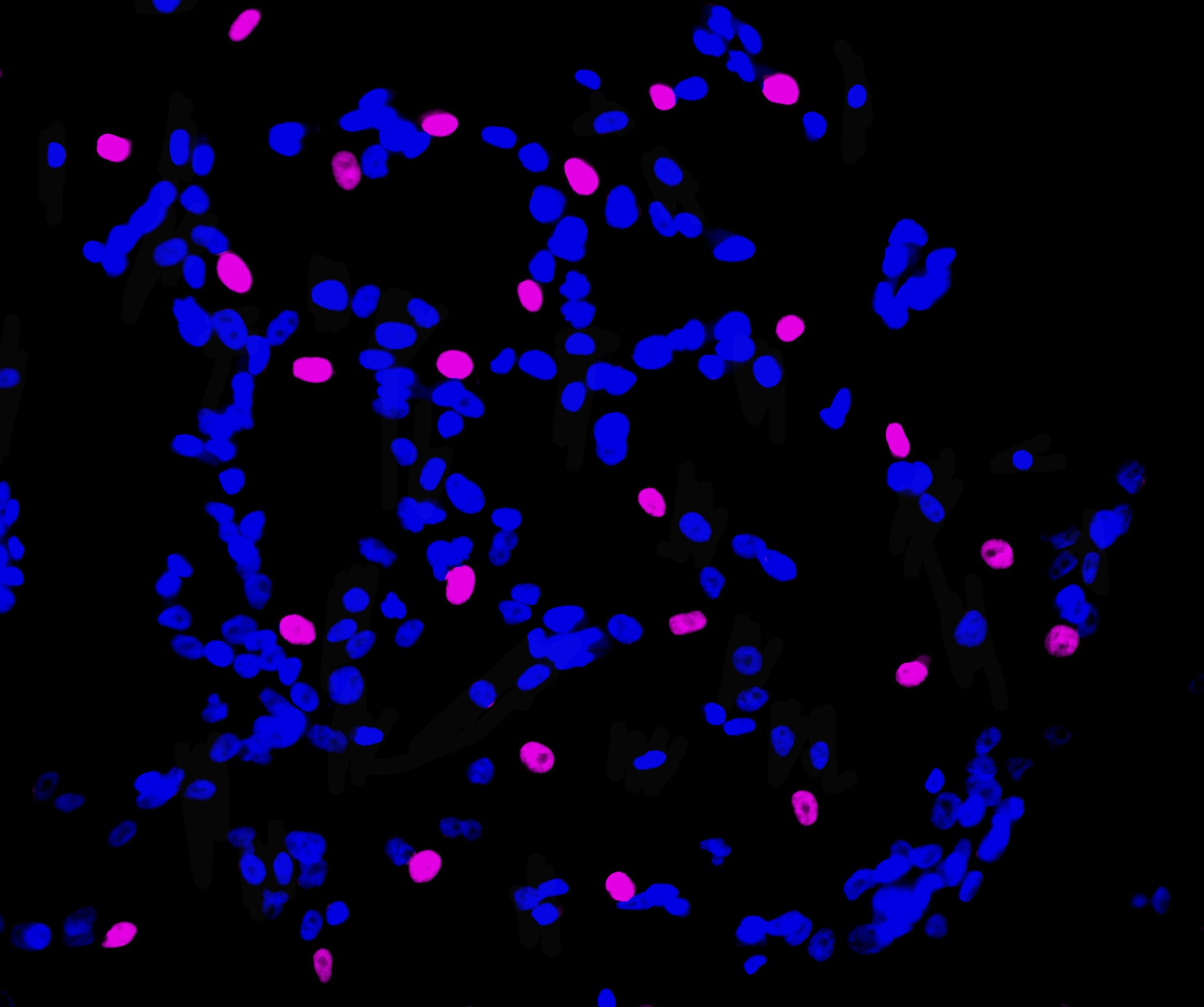

Supplement: Supplemental Information 6 — EdU assay. [file peerj-12-16868-s006.zip › Figures 8C and 8D/MKN-7-RNF144Asi-1 (2).tif]

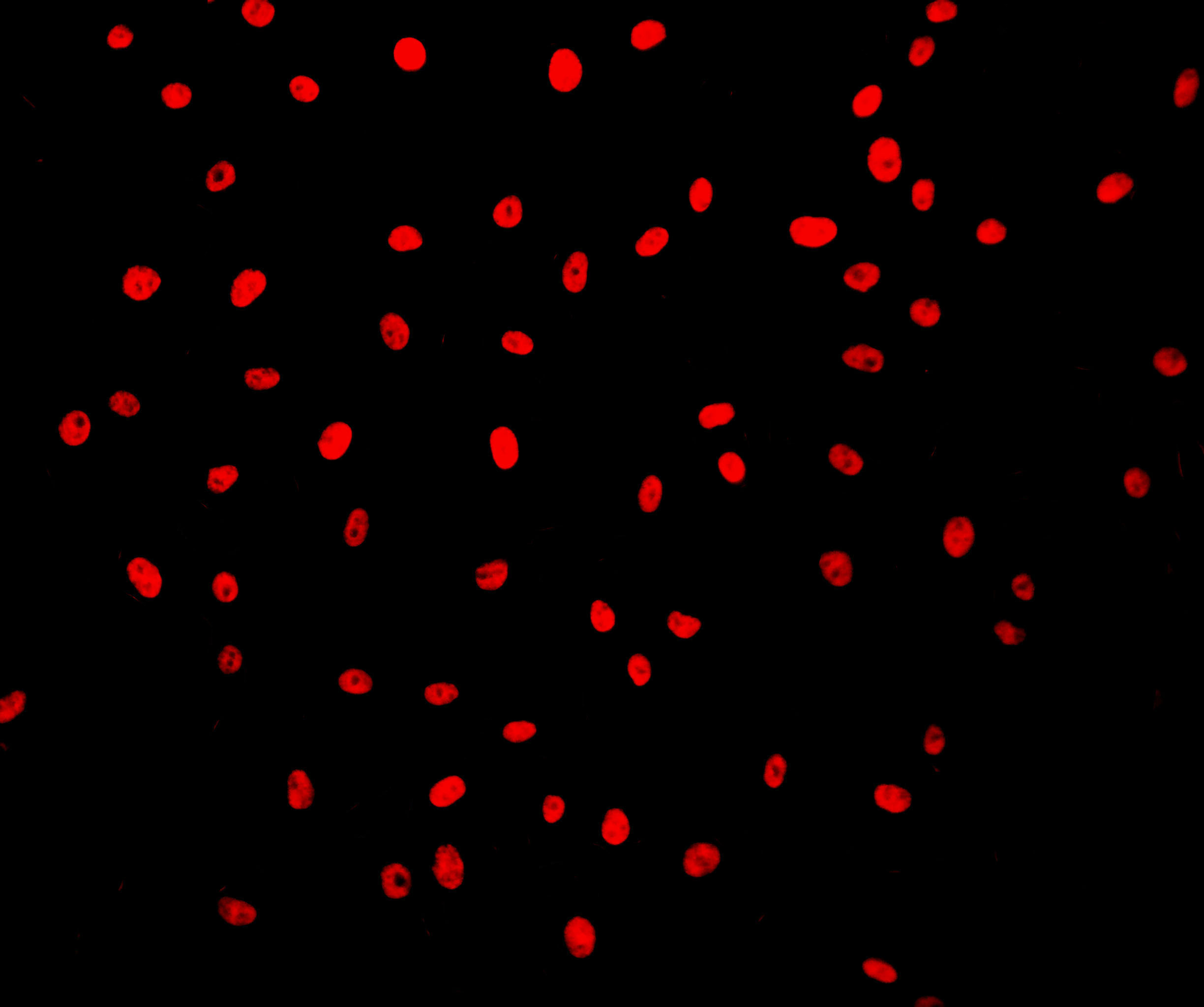

Supplement: Supplemental Information 6 — EdU assay. [file peerj-12-16868-s006.zip › Figures 8C and 8D/MKN-7-NC (2).jpg]

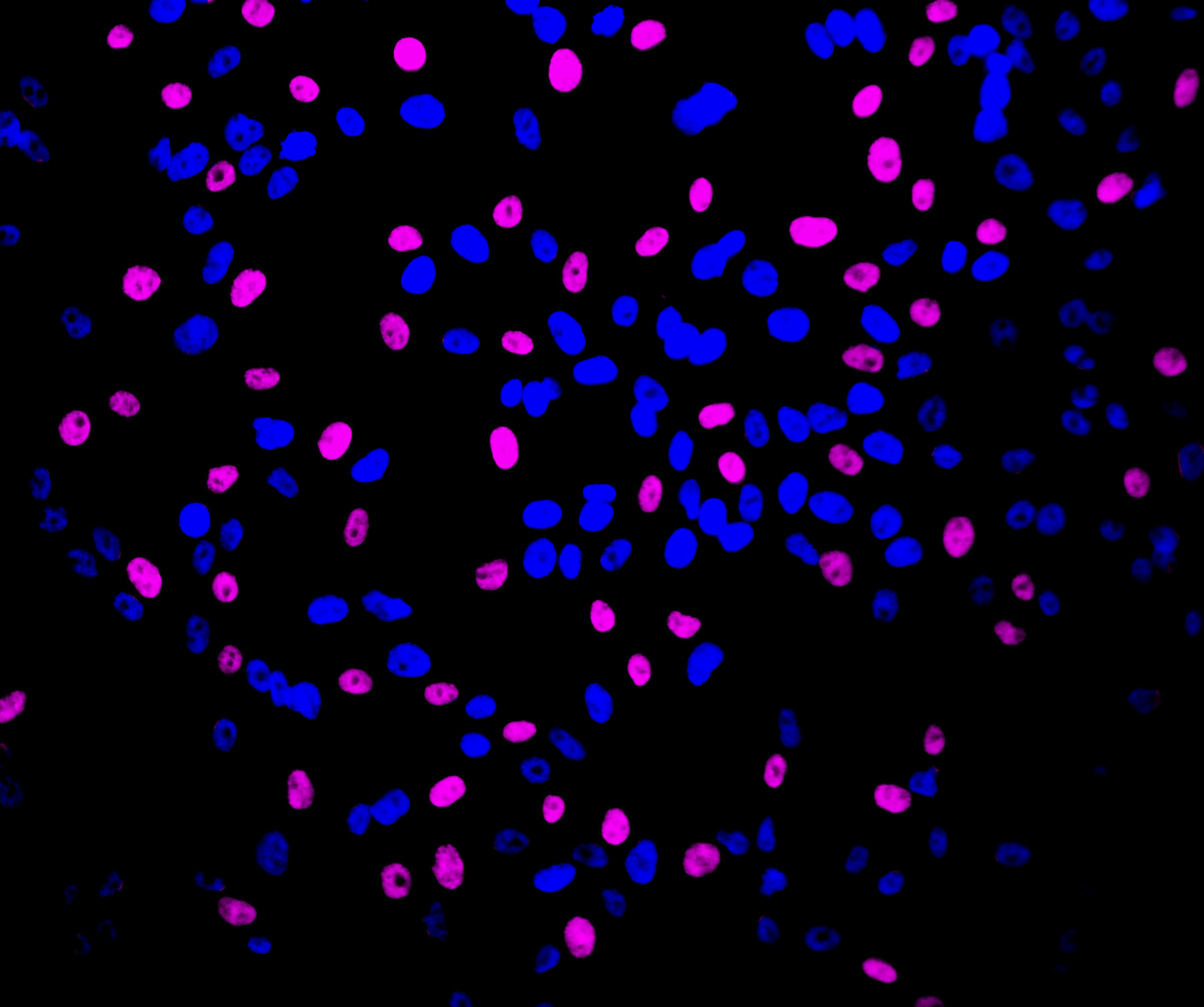

Supplement: Supplemental Information 6 — EdU assay. [file peerj-12-16868-s006.zip › Figures 8C and 8D/MKN-7-NC (2).tif]

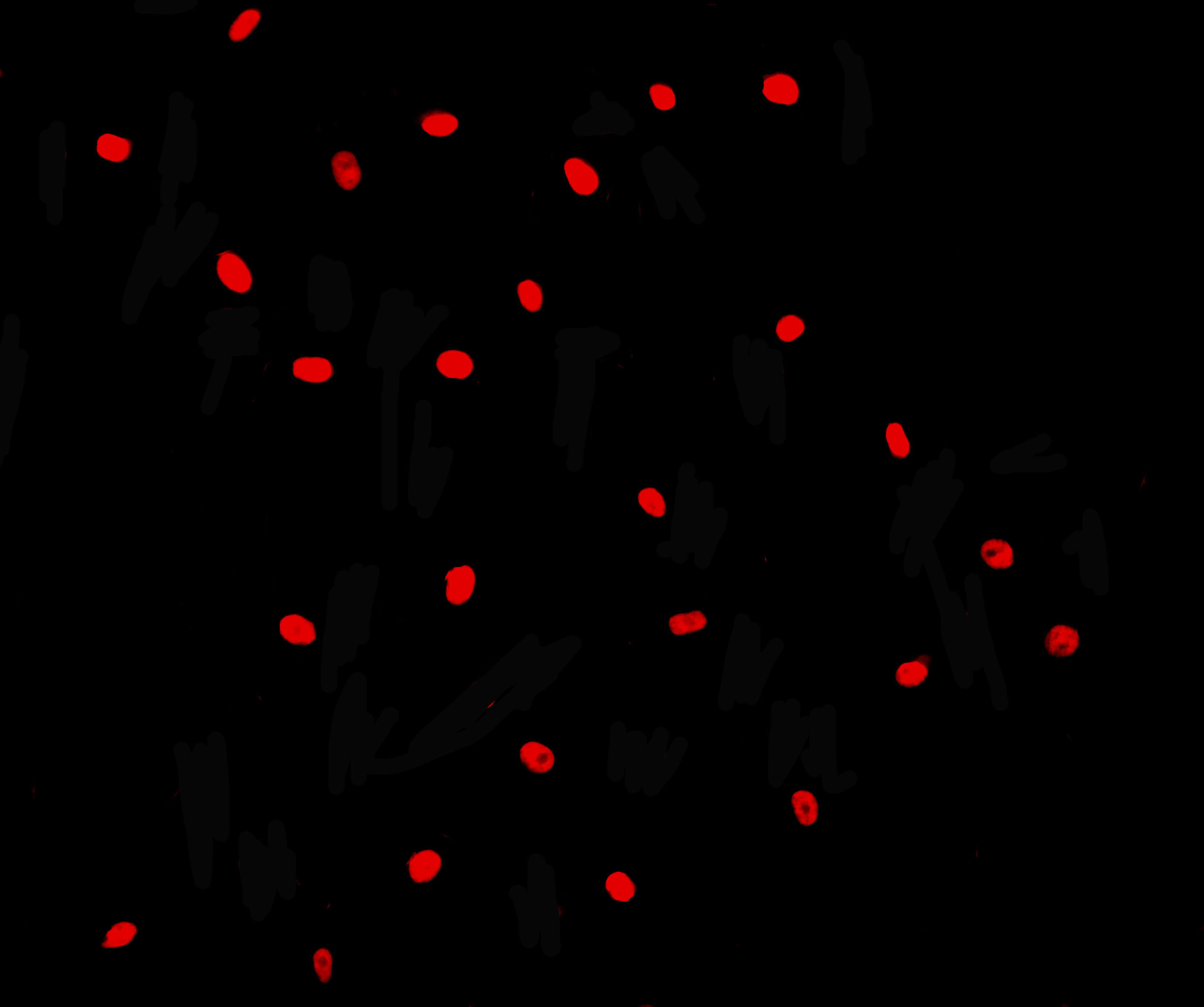

Supplement: Supplemental Information 6 — EdU assay. [file peerj-12-16868-s006.zip › Figures 8C and 8D/MKN-7-RNF144Asi-1 (2).jpg]

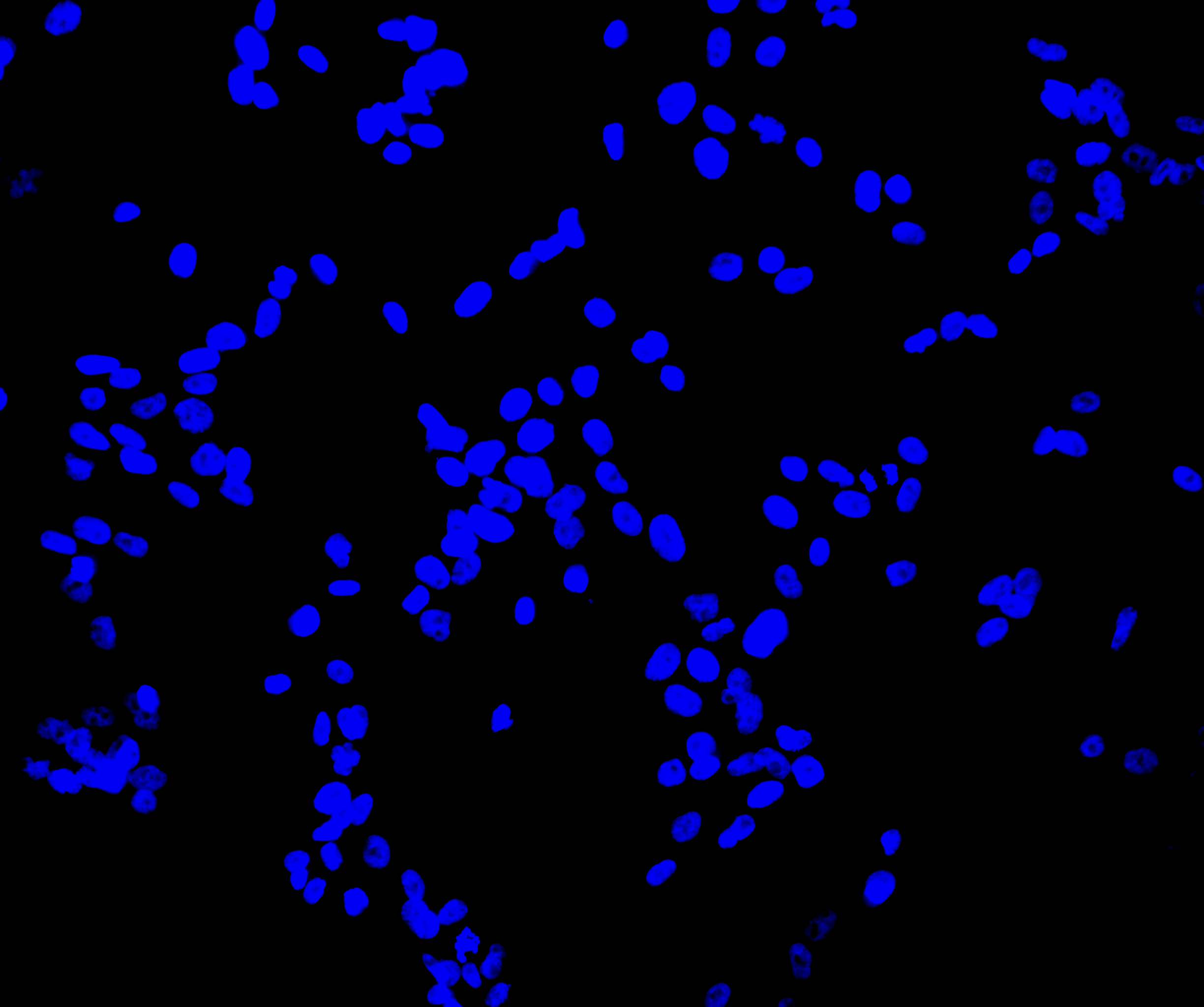

Supplement: Supplemental Information 6 — EdU assay. [file peerj-12-16868-s006.zip › Figures 8C and 8D/MKN-7-RNF144Asi-2 (2).bmp]

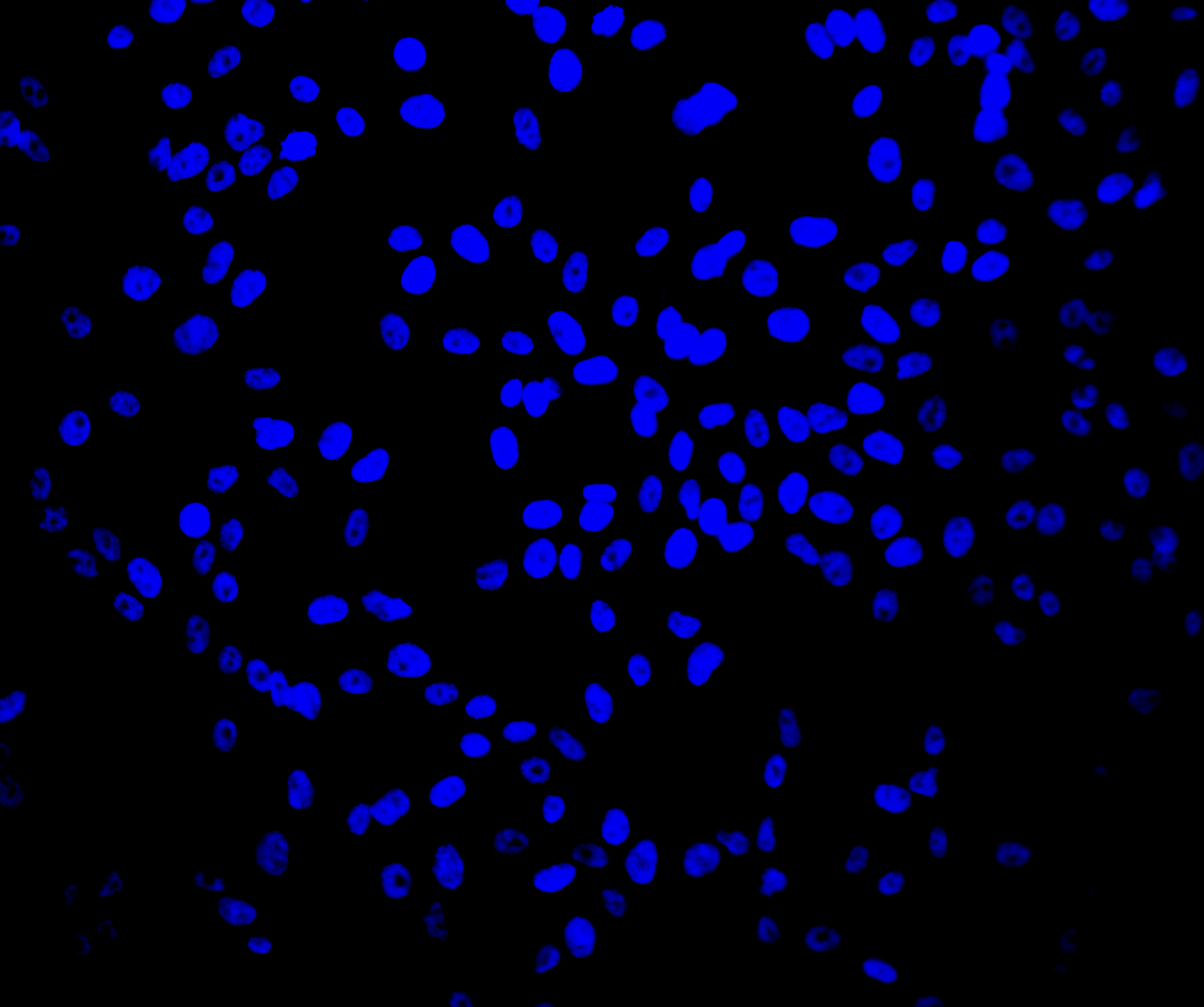

Supplement: Supplemental Information 6 — EdU assay. [file peerj-12-16868-s006.zip › Figures 8C and 8D/MKN-7-NC (2).bmp]

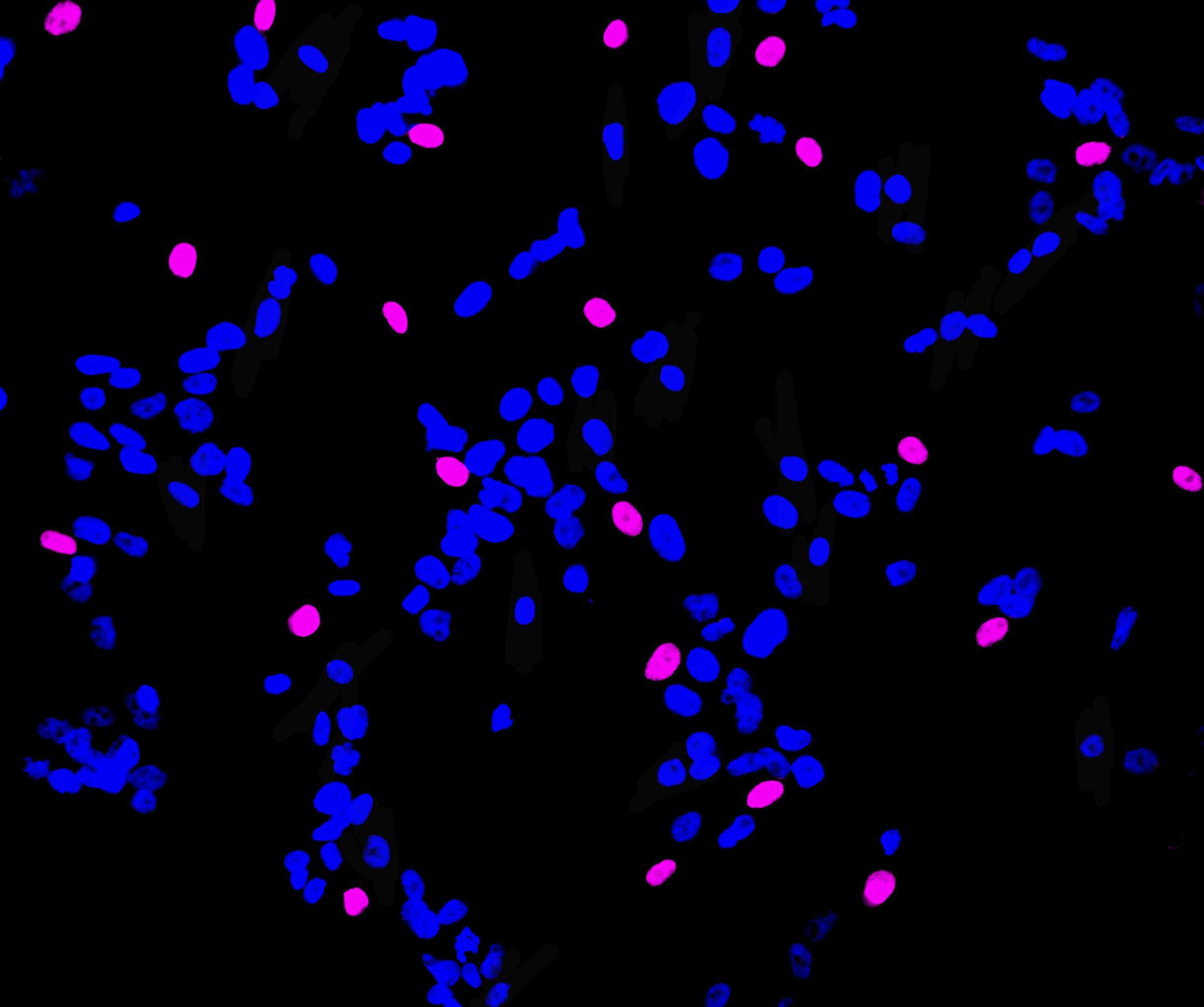

Supplement: Supplemental Information 6 — EdU assay. [file peerj-12-16868-s006.zip › Figures 8C and 8D/MKN-7-RNF144Asi-2 (2).tif]

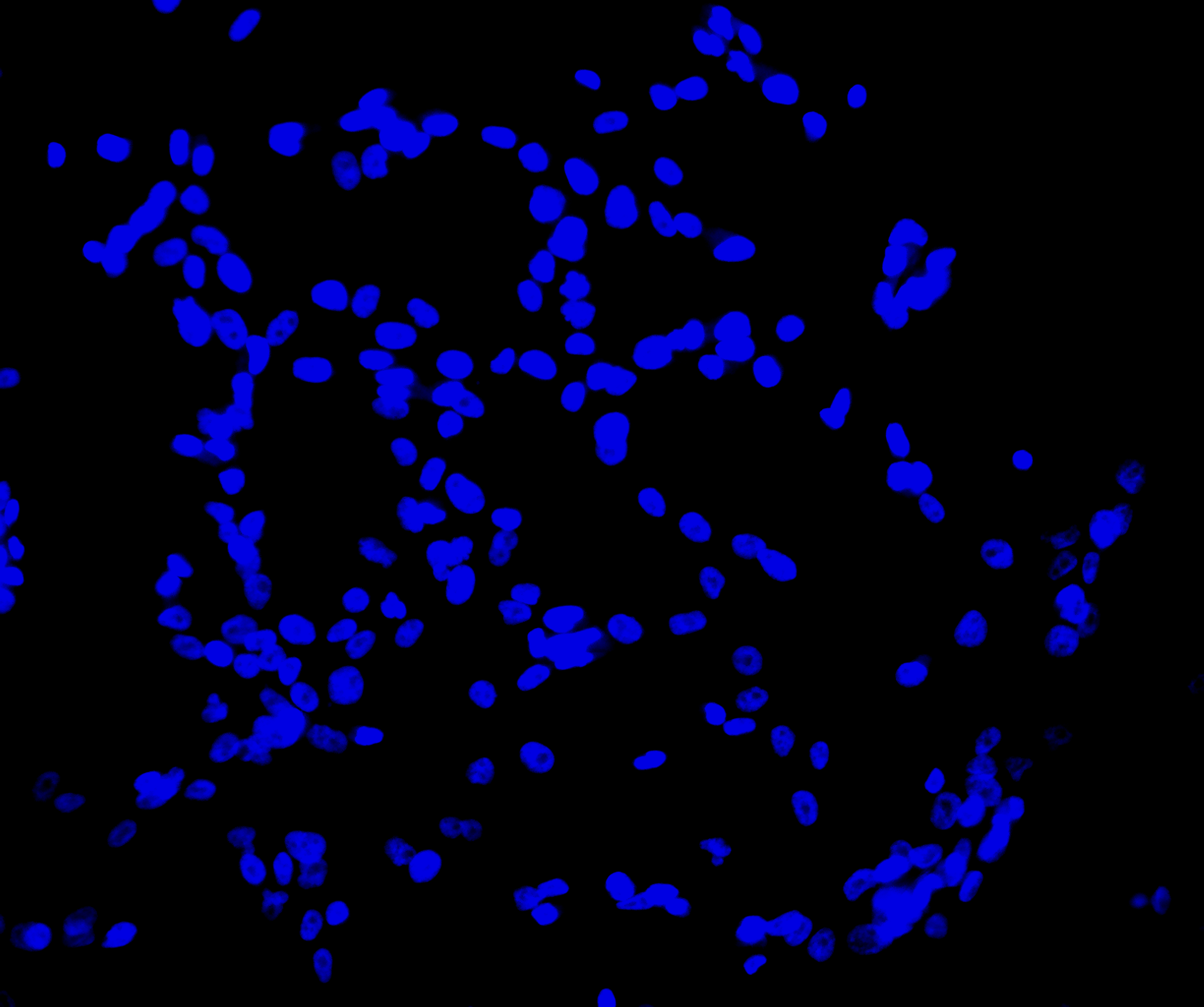

Supplement: Supplemental Information 6 — EdU assay. [file peerj-12-16868-s006.zip › Figures 8C and 8D/MKN-7-RNF144Asi-1 (2).bmp]

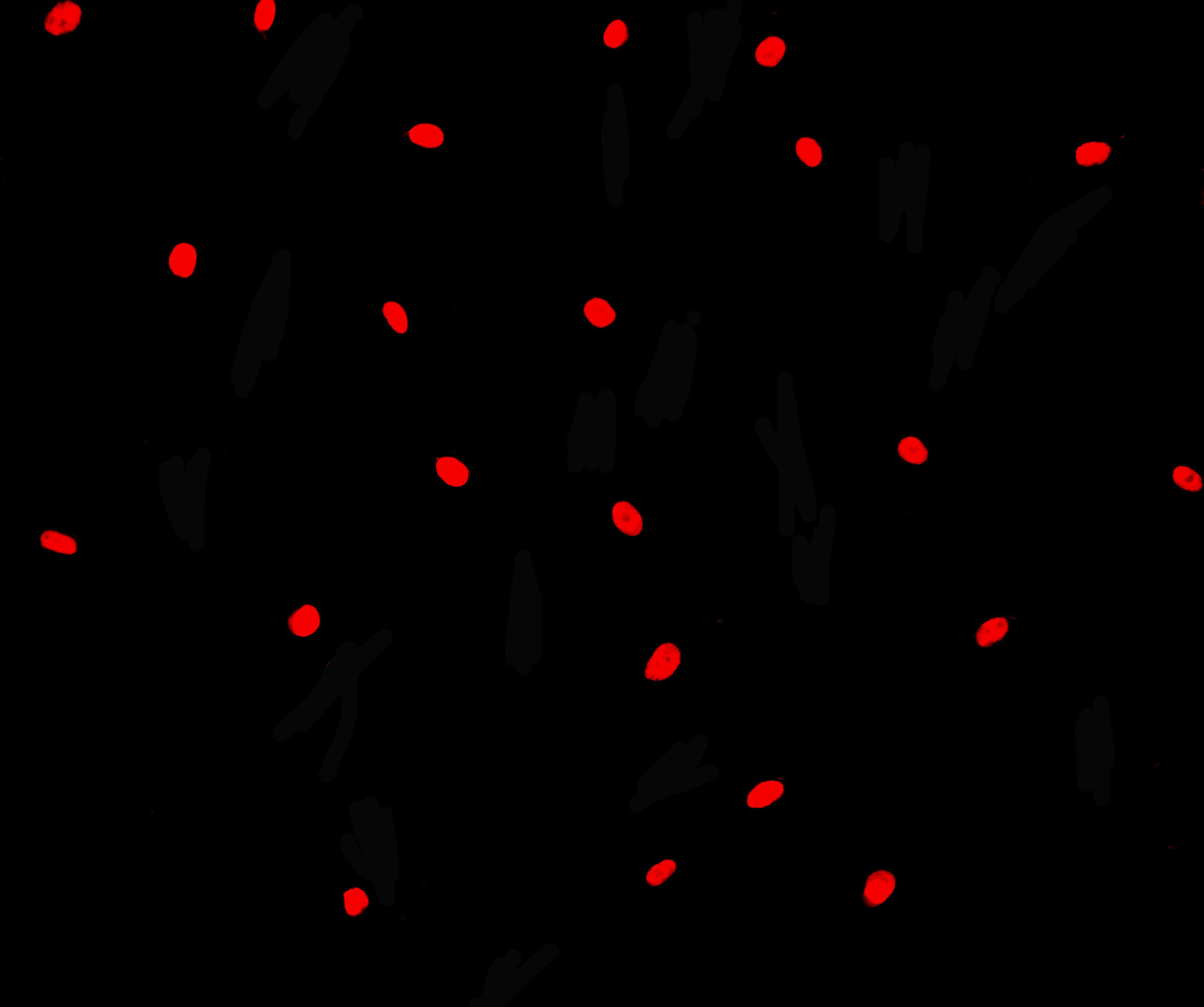

Supplement: Supplemental Information 6 — EdU assay. [file peerj-12-16868-s006.zip › Figures 8C and 8D/MKN-7-RNF144Asi-2 (2).jpg]

# Transwell

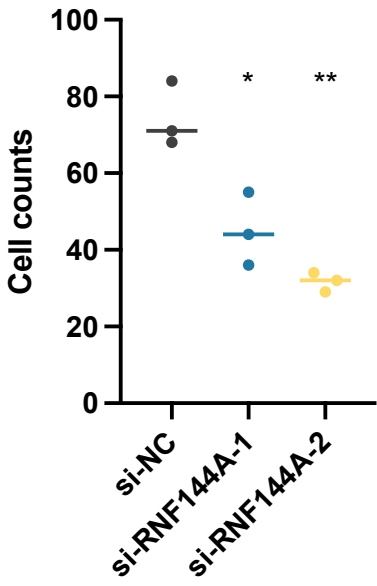

Supplement: Supplemental Information 7 — Transwell assay. [file peerj-12-16868-s007.zip › Figures 8E and 8F/Transwell.pdf]

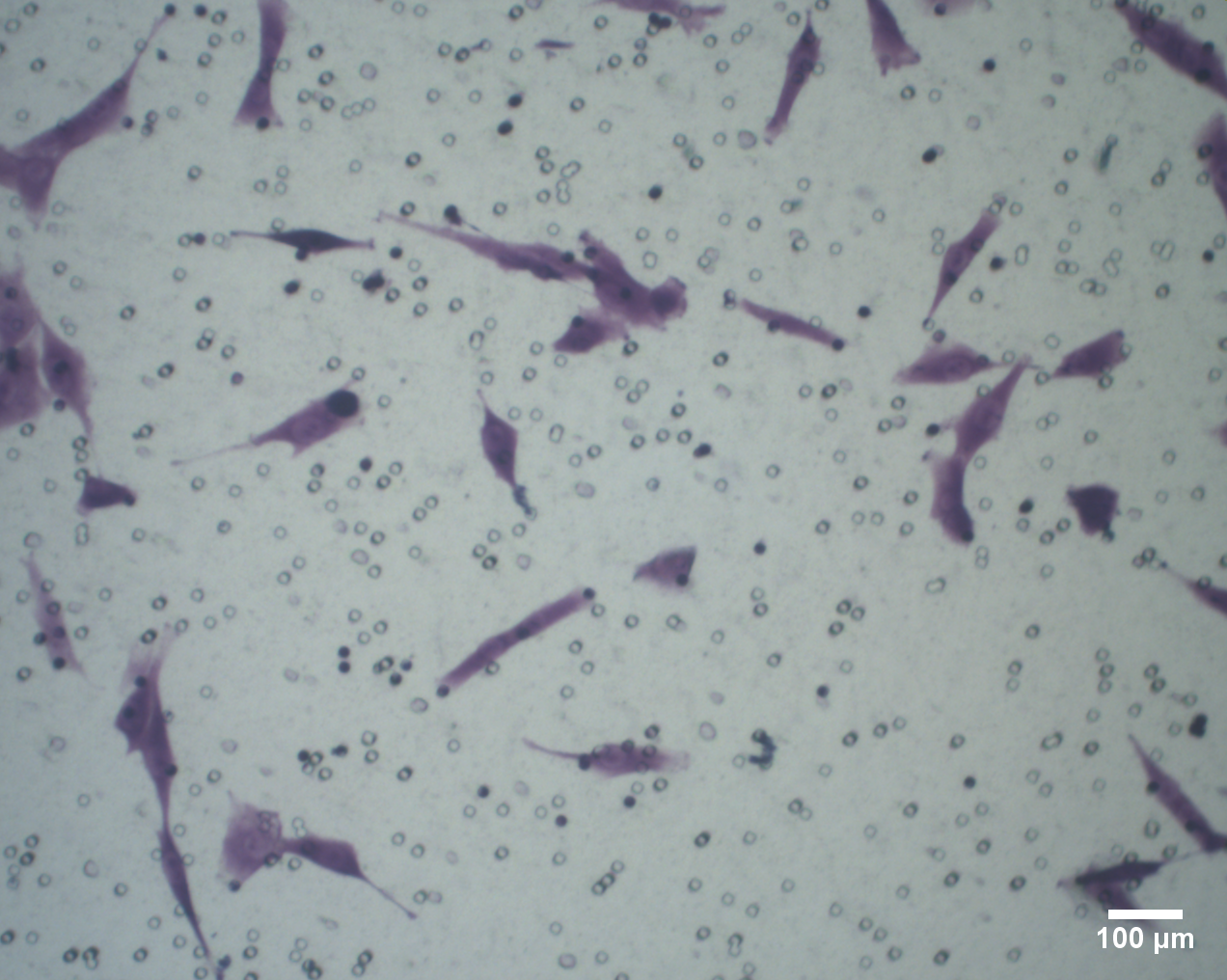

Supplement: Supplemental Information 7 — Transwell assay. [file peerj-12-16868-s007.zip › Figures 8E and 8F/MKN-7-RNF144Asi-2 (2).tif]

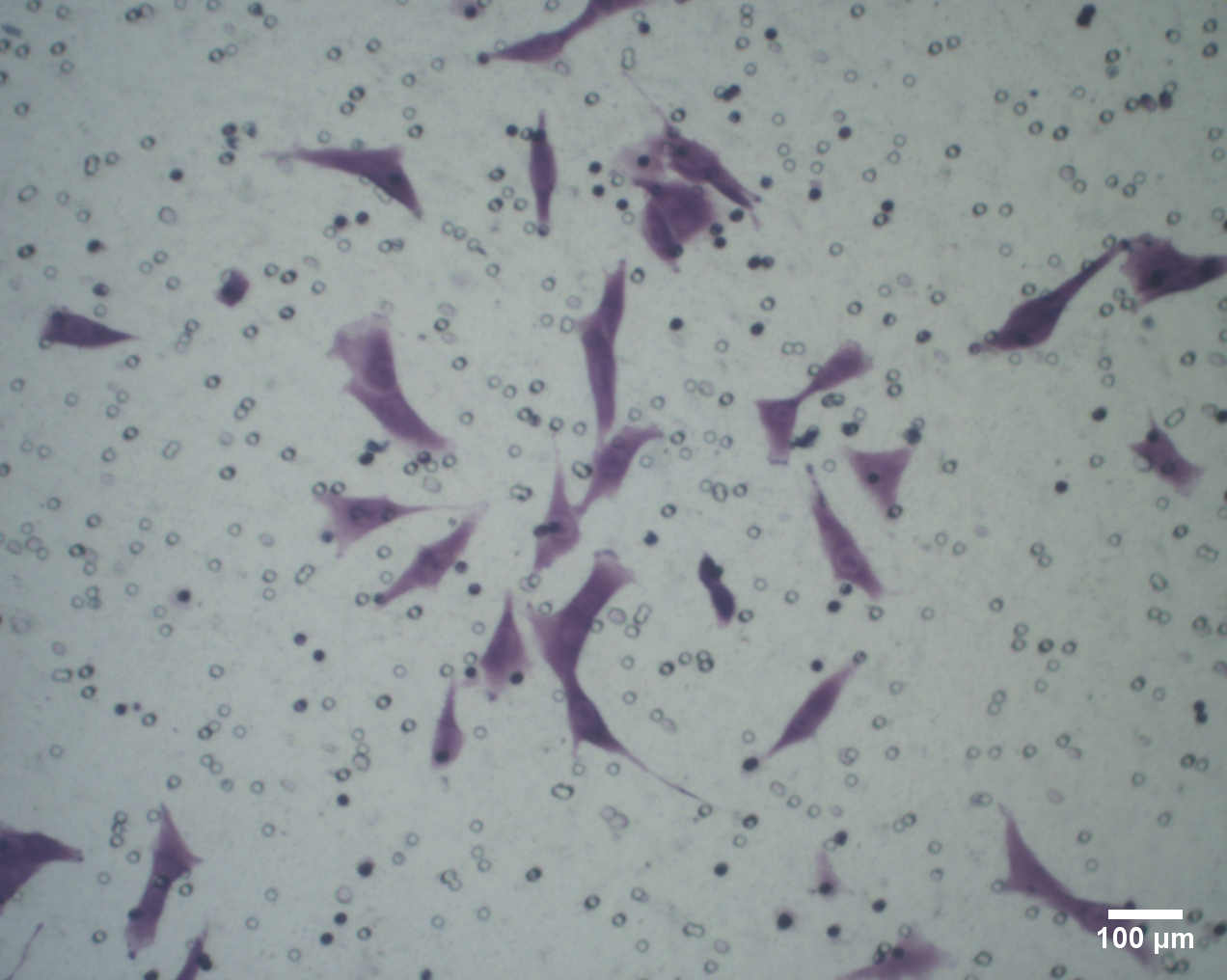

Supplement: Supplemental Information 7 — Transwell assay. [file peerj-12-16868-s007.zip › Figures 8E and 8F/MKN-7-RNF144Asi-1(2).tif]

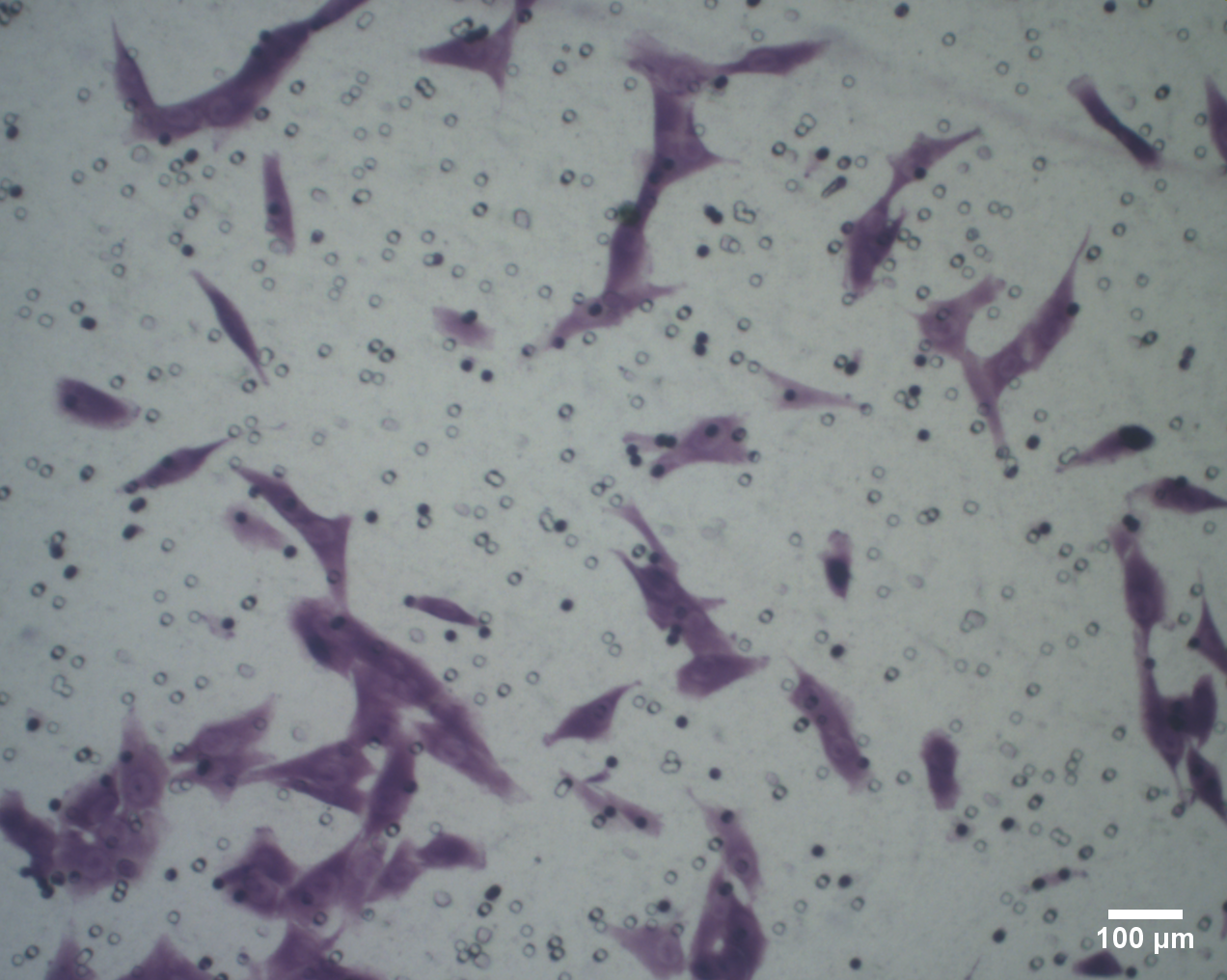

Supplement: Supplemental Information 7 — Transwell assay. [file peerj-12-16868-s007.zip › Figures 8E and 8F/MKN-7- NC(2).tif]

# M2 Macrophage

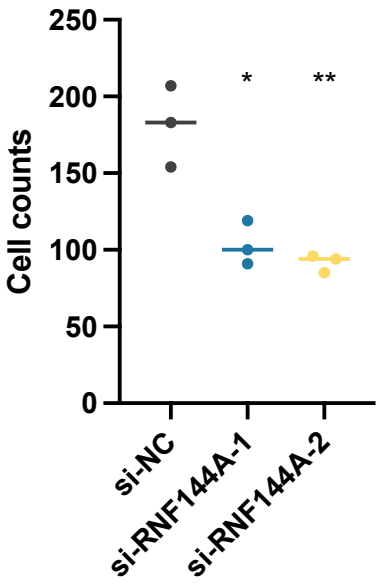

Supplement: Supplemental Information 8 — Coculture Transwell assay. [file peerj-12-16868-s008.zip › Figures 8G and 8H/M2 Macrophage.pdf]

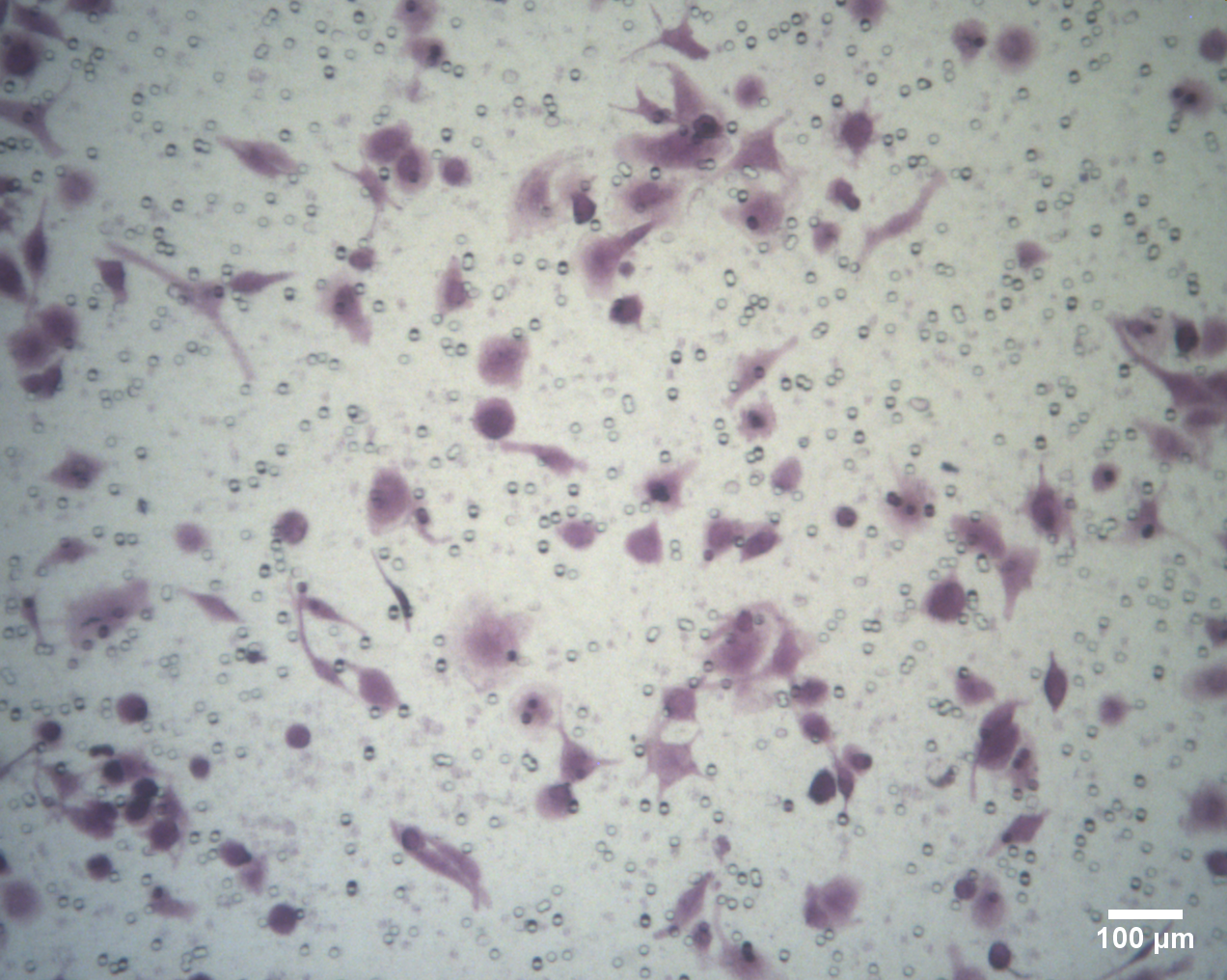

Supplement: Supplemental Information 8 — Coculture Transwell assay. [file peerj-12-16868-s008.zip › Figures 8G and 8H/MKN-7-RNF144A-2+M2 (2).tif]

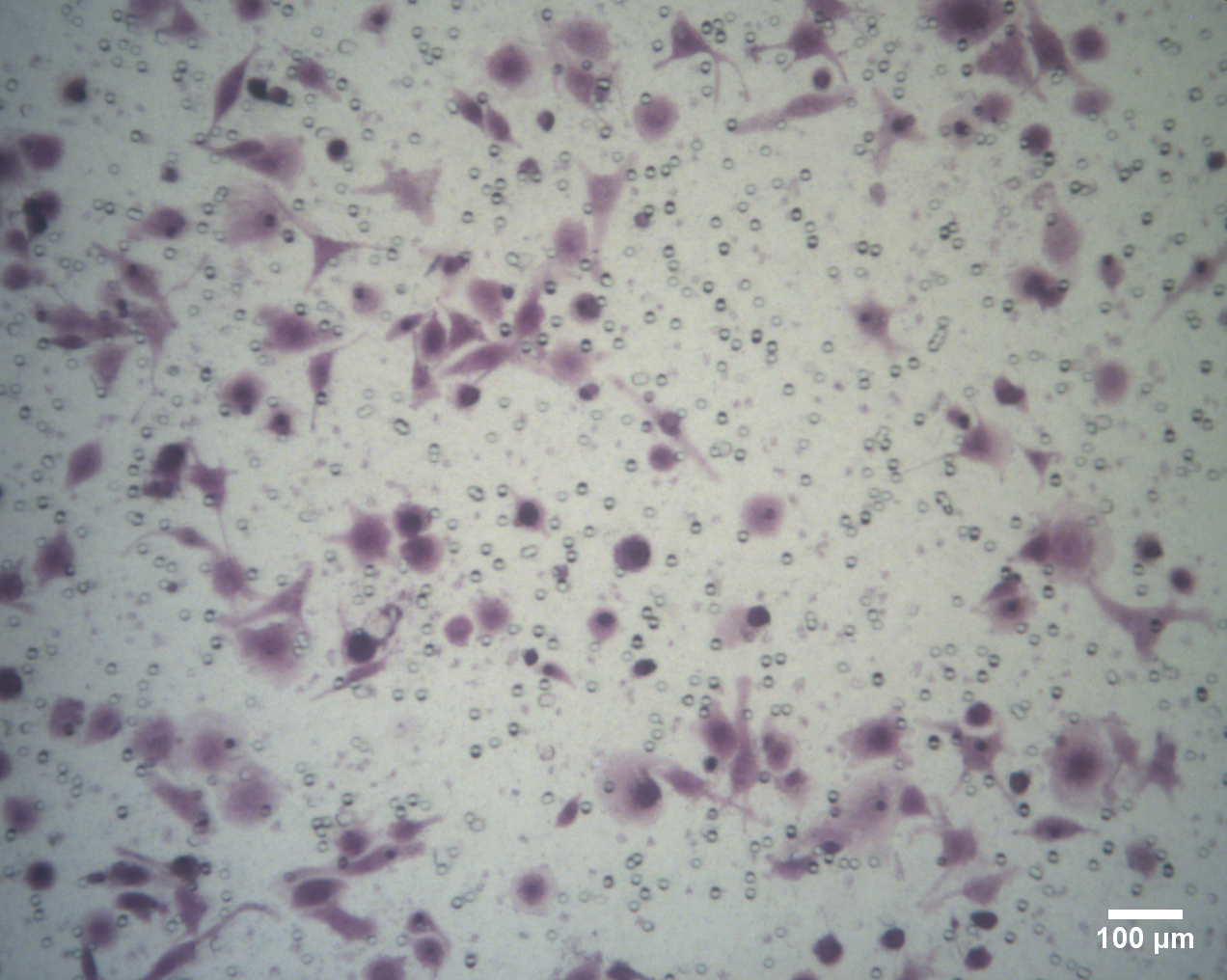

Supplement: Supplemental Information 8 — Coculture Transwell assay. [file peerj-12-16868-s008.zip › Figures 8G and 8H/MKN-7-RNF144A-1+M2 (2).tif]

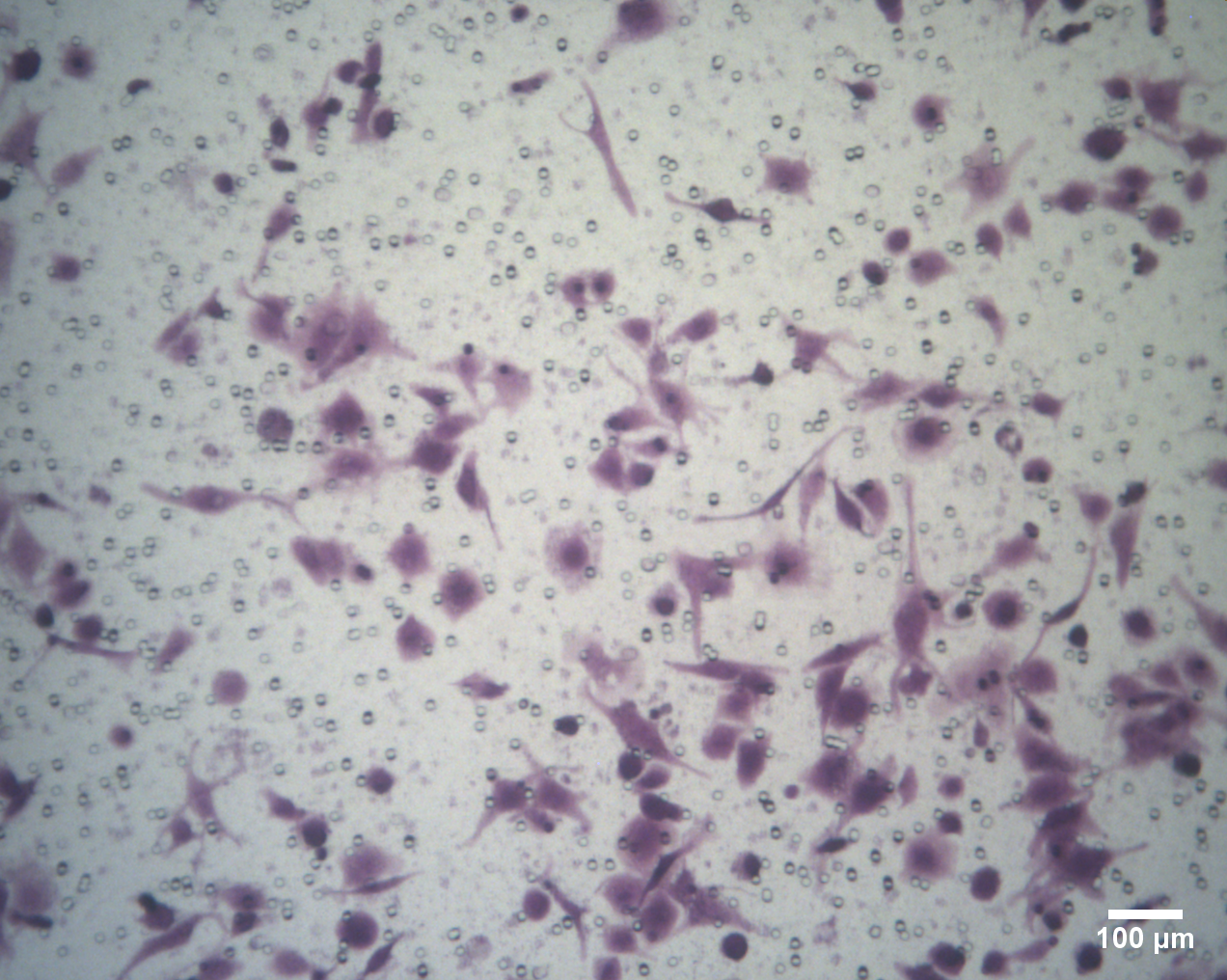

Supplement: Supplemental Information 8 — Coculture Transwell assay. [file peerj-12-16868-s008.zip › Figures 8G and 8H/MKN-7-NC+M2 (2).tif]
